# Epidemiology and Pathogenesis of Nasal NK/T-Cell Lymphoma: A Mini-Review

**DOI:** 10.1100/tsw.2011.41

**Published:** 2011-02-14

**Authors:** Katsuyuki Aozasa, Mona A. A. Zaki

**Affiliations:** Department of Pathology, Osaka University, Graduate School of Medicine, Osaka, Japan

**Keywords:** nasal lymphoma, NK/T-cell type, Epstein-Barr virus, gene abnormalities, pesticide, epidemiology

## Abstract

Nasal NK/T-cell lymphoma (NKTCL) frequently presents with necrotic, granulomatous lesions in the upper respiratory tract, and usually shows a highly aggressive clinical course. Thus, it was initially included in the clinical condition of lethal midline granuloma. Recently, the disease has been recognized as a neoplastic proliferation of NK/T cells. The disease is much more frequent in Asian and Latin American countries than in Western countries, and is universally associated with Epstein-Barr virus (EBV) infection. Analyses of gene mutations, especially p53 and c-kit, revealed the different frequencies by district. Abnormalities of other genes have also been reported. Case-control studies showed that the exposure to pesticides and chemical solvents could be causative of NKTCL. Further studies including HLA antigen typing of patients is necessary to further clarify the disease mechanism.

Rapid destruction of the nose and face (midline) by necrotic and granulomatous lesions was first described by McBride[1]. The term “lethal midline granuloma (LMG)” or “granuloma gangrenescens” were used for this condition because the disease usually showed an aggressive and lethal course. It has become evident that the LMG is composed of three different diseases, i.e., Wegener’s granulomatosis (WG), polymorphic reticulosis (PR) or midline malignant reticulosis (MMR), and malignant lymphoma[2,3]. PR and MMR are synonymous. WG is characterized by generalized necrotizing vasculitis involving both arteries and veins, and the presence of glomerulitis.

Malignant lymphoma of the nose usually shows a monomorphous proliferation of large lymphoid cells, called reticulum cells in the past. PR exhibits a polymorphous pattern of proliferation consisting of large atypical cells with mono- or multinucleus, small lymphocytes, plasma cells, benign-appearing macrophages, neutrophils, and, much less frequently, eosinophils. PR had been considered as a variant of malignant lymphoma because the disease frequently shows dissemination[2]. In 1987, Ng et al.[4] reported that tumor cells showed a positive immunoreactivity for natural killer (NK) cell marker CD56. Subsequently, it was shown that proliferating cells in PR had large granular lymphocyte morphology[5], which is characteristic of NK cells or cytotoxic T lymphocytes. There has been accumulating evidence that PR is a neoplasm of activated NK cells[6,7]; the proliferating cells usually show $CD2^+$, $CD56^+$, $CD3\varepsilon^+$, $CD7^-$, $CD16^-$, and cytotoxic granule-associated proteins$^+$, and do not exhibit rearrangement of T-

cell receptor or immunoglobulin genes (Fig. 1). Meanwhile rearrangement of T-cell receptor genes was recorded in the rare cases of PR[8,9]. In the World Health Organization classification for lymphoid neoplasms (2008), PR is termed as nasal NK/T-cell lymphoma (NKTCL). Recently, expression of the transcription factors engaged in the development of NK cells, such as T-bet and EOMES genes, in the tumor cells of NKTCL was reported[10]. Although the upper respiratory tract, especially the nasal region, is the common site of presentation, NKTCL may present in diverse extranodal sites, such as the gastrointestinal tract, skin, testes, liver, and spleen[11]. This review focuses on the epidemiology and molecular pathogenesis of nasal NKTCL.

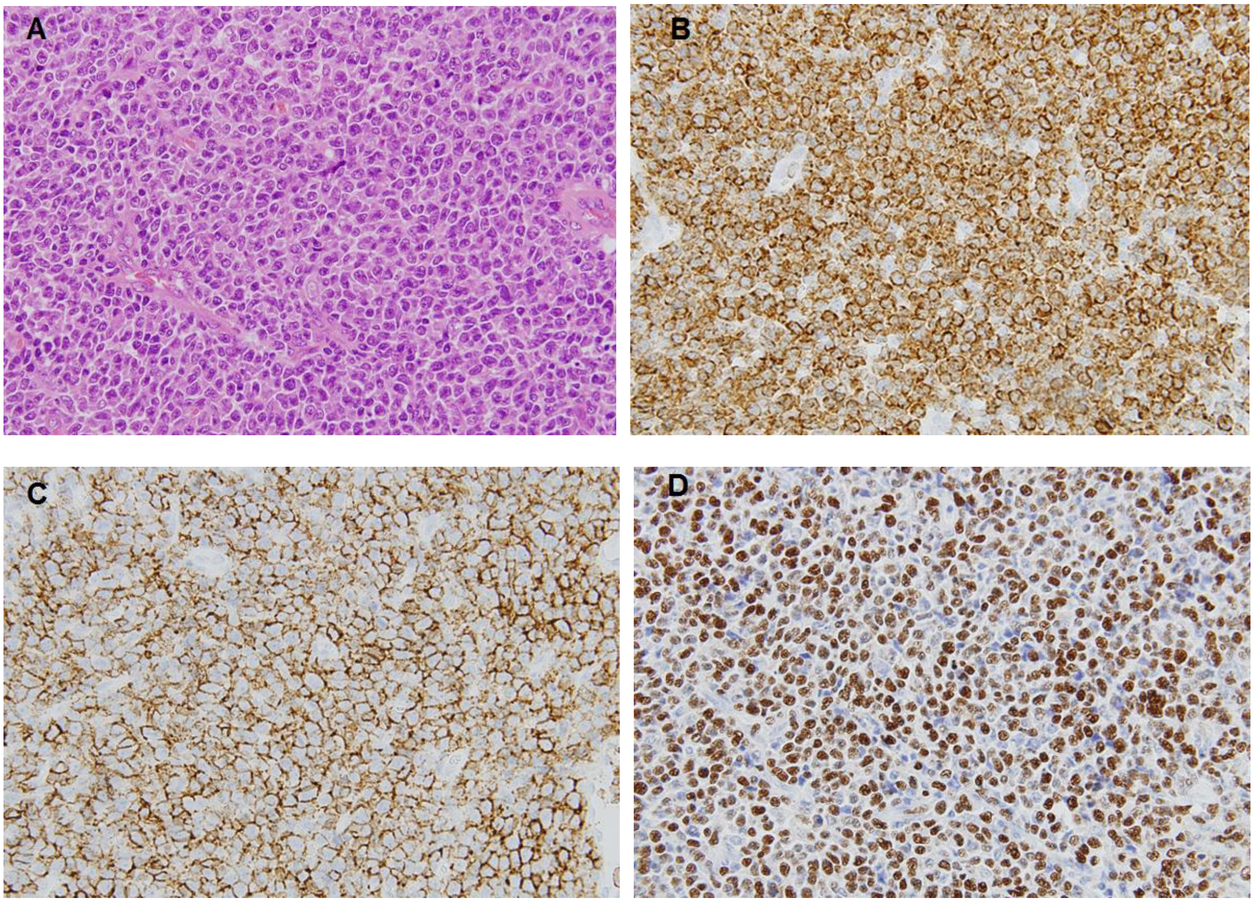

**FIGURE 1.** Large cells with irregular nuclear contour show a diffuse proliferation (A). These cells are positive for CD3 (B) and CD56 (C), and contain the Epstein-Barr virus genome (D). (A) Hematoxylin and eosin, (B,C) immunohistochemistry, (D) *in situ* hybridization with EBER probe. All ×400.

## ASSOCIATION WITH EPSTEIN-BARR VIRUS (EBV)

Previous studies revealed the constant association of NKTCL with EBV throughout the world[12,13,14]. EBV could be subtyped based on the difference in sequence of the EBNA2 region, i.e., type A and type B[15]. Almost all of the NKTCL in Korea and Japan had type A EBV[14], which is identical with the previous literature reporting that most cases of EBV-associated malignancies of immunocompetent patients in Asia had type A EBV[16]. Several studies indicated the occurrence of type B EBV in lymphomas of immunocompromised patients[17]. A predominance of type A EBV was also found in NKTCL in Malaysia[18], indicating the predominance of type A EBV in NKTCL in Asia. Borisch et al.[19] reported that three of six cases of NKTCL in Switzerland had type B EBV, although no findings suggestive of immunodeficiency were found in these patients. These findings suggest a geographic difference in the distribution of EBV subtype in the NKTCL.

The proliferating cells in NKTCL frequently express latent membrane protein (LMP) as revealed by immunohistochemistry. The cells expressing viral antigens are eliminated primarily by cytotoxic T lymphocyte (CTL) in a MHC-class-I–restricted manner[20]. Two CTL epitopes were identified in LMP-1 that are possibly pan A*02 restricted[21]. It is possible that NKTCL patients show lower frequencies of the A*02 allele compared with those in the normal population. Indeed, high-resolution genetic typing revealed a significantly lower frequency of HLA-A*0201 in NKTCL than in the normal population[22]. These findings suggest that HLA-A*0201–restricted CTL responses may function *in vivo* to suppress the development of NKTCL, or in other words, the role of EBV for NKTCL development.

## GENETIC CHANGES

Lymphoma arises from clonal expansion of lymphoid cells that are transformed by the accumulation of genetic lesions affecting oncogenes and tumor-suppressor genes. In general, the amount of samples from NKTCL lesions available for genetic analyses is small and samples usually contain massive necrotic areas. Therefore, information for genetic changes in NKTCL has been relatively limited. A recent study on the clinical samples and cell line derived from NKTCL suggests that Janus kinase signal transducers and activators of the transcription pathway might be involved in oncogenesis of NKTCL[23]. Combined gene expression profiling and immunohistochemistry on the 33 cases of NKTCL and normal NK cells revealed the overexpression of MYC, NF-κB, P53, and survivin in NKTCL cases[24].

## ALTERATIONS OF TUMOR-SUPPRESSOR GENES AND ONCOGENES

Abnormalities of p53, k-ras, c-kit, and β-catenin genes in NKTCL cases were examined by polymerase chain reaction (PCR) – single strand conformation polymorphism (SSCP) followed by a direct sequence method. P53 is a well-known tumor-suppressor gene. K-ras, c-kit, and β-catenin genes are oncogenes. In the studies for NKTCL in Asia, exons 4-8 or exons 5-8, which encompass about 90% of mutations reported in the literature[25], were examined in one institute (Table 1). The frequency of p53 mutations varied by district: high in Japan and low in Mexico, Korea, and Shenyang[26,27]. Shenyang is in northern China, adjacent to the Korean peninsula, suggesting that environmental and genetic factors might generate the differences in frequency.

**TABLE 1**
**p53 Mutations in Nasal NK/T-Cell Lymphoma**

| Country | Number of Cases | Exons Examined | Frequency of Mutations |
|---|---|---|---|
| Asia[24] | | | |
| Japan | 58 | 4-8 | 62% |
| Korea | 42 | 4-8 | 31% |
| China | | | |
| Beijing, Chengdu | 42 | 5-8 | 48% |
| Shenyang | 20 | 4-8 | 40% |
| Indonesia | 27 | 4-8 | 63% |
| Mexico[25] | 21 | 5-8 | 24% |

The development of acute leukemia or malignant lymphoma was reported in transgenic mice expressing KIT(v814)[28]. Then NKTCL in Asian countries was examined for the c-kit gene mutations. Frequency of c-kit mutations was significantly higher in China (Beijing, Chengdu) (10 of 14 cases; 71.4%) than in Japan (nine of 58 cases; 15.5%), Korea (five of 42 cases; 11.9%), and northeast China (Shenyang) (two of 20 cases; 10%)[25]. These findings suggest that location-specific differences in etiological factors might cause specific mutations in the c-kit gene.

## FAS GENE MUTATIONS

Fas (Apo-1/CD95) is a 45-kDa membrane protein belonging to the tumor necrosis factor receptor family and mediates programmed cell death (apoptosis) through binding of the Fas ligand (Fas L)[29]. Fas consists of 325 amino acids with a single transmembrane domain, including signal peptide. The 80-amino-acid portion in the cytoplasm, designated as a death signaling domain, is essential for the apoptotic signal transduction. NKTCL frequently coexpress Fas and Fas ligand (Fas L), but the tumor cells seldom undergo apoptosis. Some mechanisms for resistance to Fas/Fas L-induced apoptosis might work in the development of NKTCL, thus Fas gene mutations could be one of the mechanisms. Two reports support this notion: NKTCL cells in nine of 15 (60%) cases[30] and seven of 14 (50%) cases[31] showed mutations of the Fas gene. Mouse T-cell lymphoma cells transfected with mutated Fas genes were resistant to apoptosis[31], indicating the mutations to be a loss-of-function mutation. These findings suggest that accumulation of lymphoid cells with Fas mutations provides a basis for the development of NKTCL.

## EPIDEMIOLOGICAL FEATURES

Cases of NKTCL, called PR in the past, are occasionally encountered in Japan. When one of the authors (KA) visited the U.S. in 1987, he noticed that the disease was quite rare in the U.S., but they had consultation cases from Peru. Because Japan and part of Peru also have Mongolian ethnic populations, it was postulated that the Mongolian group might be much more susceptible to this disease. Then the authors started to examine the frequency of NKTCL in East Asian countries during the period from 1987 to 1993; the frequency of each disease constituting LMG is shown as the frequency per 100,000 patients who visited the Ear-Nose-Throat clinics. All of the histological sections of LMG were reviewed by one of the authors (KA). Frequency of PR ranged from eight to 40.8. That in the Institute of Laryngology and Otology, London (1966–1987) was four, showing two to 10 times higher frequencies of PR in the East Asian countries[32,33]. The disease is also rare in Europe. The patients with NKTCL also seem to be clustered in Latin American countries and Indonesia. Information from other parts of the world such as Africa, the Middle and Near East, and Russia is helpful to elucidate the etiology of this disease.

## LIFE STYLE AND ENVIRONMENTAL FACTORS

Recently, the first case of familial NKTCL affecting a father and one of his six children was reported[34]. They used large amounts of pesticide in a greenhouse. An increase in the risk of developing non-Hodgkin’s lymphomas (NHL) among individuals exposed to pesticides was reported[35,36]. Association of pesticides with a risk of developing t(14;18)-positive NHL, but not t(14;18)-negative NHL, was reported[37]. These findings might raise the possibility of a causative role for some genetic, environmental, and life style factors in NKTCL development. Therefore, an epidemiological study to elucidate whether socioenvironmental ambient factors contribute to the development of NKTCL was conducted as a collaborative study of Japan, Korea, and China (Table 2)[38]. The odds ratio (OR) of NKTCL was 4.15 (95% confidence interval [CI], 1.74–9.37) for farmers, 2.81 (CI, 1.49–5.29) for producers of crops, and 4.01 (CI, 1.99–8.09) for pesticide users. The OR for crop producers who minimized

**TABLE 2**
**Risk of Nasal NK/T-Cell Lymphoma in Relation to Cultivation of Crops and Pesticide Use[37]**

| | Odds Ratio (OR)[a] | Number of Cases (n = 88) | Number of Cases (n = 305) | 95%CI | |
|---|---|---|---|---|---|
| | | | | Lower | Upper |
| Cultivation of crops | | | | | |
| At present | 2.81 | 27 | 36 | 1.49 | 5.29 |
| More than 5 years | 5.08 | 24 | 19 | 2.47 | 10.43 |
| Pesticides | | | | | |
| Users | 4.01 | 23 | 23 | 1.99 | 8.09 |
| Type of pesticide | | | | | |
| Herbicide | 3.17 | 13 | 16 | 1.36 | 7.38 |
| Insecticide | 3.45 | 20 | 21 | 1.67 | 7.13 |
| Fungicide | 6.05 | 10 | 6 | 1.98 | 18.46 |
| Precautions | | | | | |
| Gloves used | 3.30 | 10 | 11 | 1.28 | 8.54 |
| Gloves not used | 4.76 | 13 | 12 | 1.93 | 11.72 |
| Mask used | 5.44 | 14 | 10 | 2.20 | 13.47 |
| Mask not used | 2.82 | 9 | 13 | 1.08 | 7.37 |
| Glasses used | 1.18 | 1 | 3 | 0.11 | 12.13 |
| Glasses not used | 4.52 | 22 | 20 | 2.17 | 9.42 |
| Sprinkling downward, attended | 2.20 | 9 | 16 | 0.88 | 5.53 |
| Sprinkling downward, not attended | 8.45 | 14 | 7 | 3.01 | 23.70 |

[a] Adjusted for age (<40, 40–59, >60), sex (male/female), and country (Japan, Korea, and China).

their exposure to pesticides by using gloves and glasses, and sprinkling downwind at the time of pesticide use, were 3.30 (CI,1.28–8.54), 1.18 (CI,0.11–12.13), and 2.20 (CI, 0.88–5.53), respectively, which were lower than those for producers who did not take these precautions. Taken together, exposures to pesticides and chemical solvents could be causative factors for NKTCL.

## CONCLUSION

The clinical course of patients with NKTCL is highly aggressive. Therefore, clarification of risk factors for disease development is especially important in order to establish a strategy for disease prevention. Because EBV infection and pesticides could be risk factors for NKTCL, investigation on effects of pesticides for EBV activation is needed. A correlation of exposure to certain pesticides and organochlorines with increased titers of antibodies to EBV was reported[39]. Patients with NKTCL seem to be clustered in Asian and Latin American countries; therefore, some genetic factors might be involved in the disease development. There have been no reports describing an association of human T-lymphotropic virus (HTLV)-I with NKTCL. We examined the presence of serum antibody for HTLV-I, revealing negativity (data not published). Further studies including HLA antigen typing of patients might provide a clue for understanding the disease development.

## ACKNOWLEDGMENTS

This work is supported in part by grants (1639015, 18014015) from the Ministry of Education, Culture, Sports, Science and Technology, Japan.
